# Adrenal crisis as a reversible etiology of heart failure with preserved ejection fraction: insights from a case series

**DOI:** 10.3389/fcvm.2025.1623782

**Published:** 2025-11-04

**Authors:** Mengmei Li, Ruicai Shan, Ling Song, Chengsen Zhang

**Affiliations:** ^1^ Department of Emergency, Qingdao Central Hospital, University of Health and Rehabilitation Sciences, Qingdao, China; ^2^ Department of Abdominal Ultrasound, Qingdao Central Hospital, University of Health and Rehabilitation Sciences, Qingdao, China; ^3^ Department of Outpatient, Qingdao Municipal Hospital, Qingdao, China

**Keywords:** HFpEF, adrenal crisis, myocardial infarction, adrenal insufficiency, cancer therapy

## Abstract

**Background:**

Adrenal crisis, characterized by acute cortisol deficiency, is a rare, life-threatening condition that can precipitate cardiovascular collapse and heart failure (HF). Its role in HF with preserved ejection fraction (HFpEF) is underrecognized, particularly in cancer patients receiving therapies that impair adrenal function. This case series examines the clinical features, management, and outcomes of HFpEF induced by adrenal crisis, emphasizing early diagnosis and treatment.

**Methods:**

We retrospectively analyzed four patients diagnosed with HFpEF secondary to adrenal crisis between January 2022 and January 2025 at Qingdao Central Hospital and Qingdao Municipal Hospital. Inclusion criteria included clinical evidence of adrenal crisis (low cortisol, hypotension, steroid responsiveness) and echocardiographic confirmation of HFpEF (EF ≥50%). Data on demographics, clinical presentation, laboratory findings, echocardiography, and outcomes were analyzed descriptively.

**Results:**

The cohort comprised three males and one female (aged 41–77 years), all with HFpEF (EF 50%–60%). Two presented with myocardial infarction (one NSTEMI, one STEMI), and two had malignancy with adrenal metastasis (renal, lung). Three exhibited hypotension. Initial BNP levels ranged from 518.93–619.13 pg/mL, decreasing to 108.06–287.63 pg/mL pre-discharge after hormone replacement therapy and HF management. Mean EF improved by 1.75% (range: 0%–3%) at one-month follow-up, with BNP further declining to 20.36–177.24 pg/mL. All patients achieved symptom resolution with no recurrence reported.

**Conclusion:**

Adrenal crisis is a rare, reversible etiology of HFpEF in patients with diverse underlying conditions, potentially including those with cancer-related adrenal dysfunction or prior therapies. Prompt steroid therapy appears to improve cardiac function and outcomes, suggesting a need for heightened awareness and consideration of adrenal screening in at-risk populations, such as those with malignancy, tuberculosis, or other causes of adrenal insufficiency. Larger studies are needed to confirm these preliminary findings and establish the prevalence of this etiology across different subpopulations.

## Introduction

Heart failure (HF) is a complex syndrome with diverse etiologies, including ischemic heart disease, hypertension, and valvular disorders, yet endocrine causes such as adrenal crisis remain underrecognized (1). HF with preserved ejection fraction (HFpEF), accounting for approximately half of all HF cases, is increasingly prevalent and characterized by impaired diastolic function, systemic inflammation, and multiorgan stressors, distinct from the systolic dysfunction typical of HF with reduced ejection fraction (HFrEF) (2). While traditional cardiovascular risk factors dominate HFpEF research, rare endocrine triggers like adrenal crisis—marked by acute cortisol deficiency—may disproportionately contribute to this phenotype by disrupting vascular tone, volume status, and myocardial performance (3, 4). Adrenal crisis, a life-threatening condition, is well-documented for causing hypotension and shock, but its role as a precipitant of HF, particularly HFpEF, is rare and poorly understood.

The adrenal glands are frequent sites of metastasis from cancers such as lung, breast, and renal malignancies, with studies reporting adrenal insufficiency in up to 50% of patients with bilateral adrenal metastases, increasing the risk of adrenal crisis (5–8). Modern cancer therapies—chemotherapy, radiotherapy, and immunotherapy—further elevate this risk by causing direct adrenal damage or secondary hypophysitis (9, 10). In patients with underlying cardiac vulnerabilities, such as those exposed to cardiotoxic treatments, adrenal crisis may exacerbate myocardial dysfunction, potentially presenting as acute HFpEF rather than HFrEF due to its effects on preload reduction and systemic inflammation rather than overt systolic failure (11, 12). Despite its severity, this interaction is infrequently reported, and clinical data on its presentation and management are limited.

This case series addresses this gap by exploring the clinical characteristics, treatment strategies, and outcomes of HFpEF induced by adrenal crisis. We hypothesize that adrenal crisis is a reversible HFpEF etiology, in patients with various underlying conditions, such as cancer-related adrenal dysfunction, and that early steroid therapy can improve cardiac function and survival. By analyzing four cases, we aim to enhance recognition of this rare condition, inform multidisciplinary management, and underscore the need for adrenal function screening in at-risk patients, potentially improving outcomes at the critical intersection of endocrinology, cardiology, and oncology.

## Methods

This study was a retrospective case series conducted at Qingdao Central Hospital and Qingdao Municipal Hospital, both affiliated with the University of Health and Rehabilitation Sciences, between January 2022 and January 2025. We aimed to characterize the clinical features, management, and outcomes of heart failure (HF) induced by adrenal crisis in a small cohort of patients. The study was approved by the Qingdao Central Hospital Ethics Committee, and informed consent was waived due to the retrospective design and use of de-identified data, in compliance with Chinese national regulations and the Declaration of Helsinki.

Patients were identified through a two-step process. First, electronic medical records (EMRs) were screened using International Classification of Diseases (ICD-10) codes for adrenal insufficiency (E27.1, E27.2) and heart failure (I50) to identify potential cases. Subsequently, these records were further evaluated to confirm eligibility based on the following criteria: (1) clinical diagnosis of adrenal crisis, defined by low serum cortisol levels (<5 µg/dl), hypotension (systolic blood pressure <90 mmHg) or symptoms (e.g., fatigue, nausea) responsive to corticosteroid therapy, and supporting laboratory findings (e.g., hyponatremia, elevated ACTH in primary insufficiency, or ACTH stimulation test results when available); (2) echocardiographic evidence of HF with preserved ejection fraction (HFpEF), classified as EF ≥50% per European Society of Cardiology guidelines; and (3) treatment and follow-up data available during the study period. Adrenal crisis was confirmed in all cases by morning cortisol levels. Exclusion criteria included: (1) HF attributable to primary cardiovascular causes, such as significant coronary artery disease, severe valvular disease (ruled out by echocardiography), or acute myocardial infarction independent of adrenal insufficiency; (2) incomplete medical records; (3) alternative endocrine etiologies (e.g., thyroid storm); and (4) HF with reduced ejection fraction (HFrEF; EF <40%).

One patient with HFrEF was identified during screening but excluded due to the focus on HFpEF. Four patients meeting these criteria were included. Data were collected on demographics, clinical presentation, laboratory results (e.g., troponin I, BNP, cortisol, ACTH), echocardiographic parameters (e.g., EF, left ventricular dimensions), underlying conditions (e.g., cancer history, therapy), treatment regimens, and outcomes at discharge and one-month follow-up. Data were extracted by two investigators and cross-verified for accuracy. Descriptive analysis was performed, summarizing findings as individual case narratives and aggregated trends (e.g., mean changes in BNP and EF). No statistical comparisons were conducted due to the small sample size.

## Results

This retrospective case series included four patients diagnosed with heart failure with preserved ejection fraction (HFpEF) secondary to adrenal crisis between January 2022 and January 2025. Below, we summarize clinical characteristics, treatment responses, and outcomes, with detailed data presented in Tables 1.

**Table 1:** Clinical characteristics and outcomes of patients with heart failure induced by adrenal crisis.

Parameter	Case 1	Case 2	Case 3	Case 4
Demographics				
Age (years)	75	77	51	41
Sex	Female	Male	Male	Male
Prior AI Dignosis	No	No	Yes (discontinued prednisone)	No
Precipitant for Adrenal Crisis	Pneumonia	Adrenal metastasis (no infection)	Pneumonia	Tuberculosis
Clinical Presentation				
HF Type	HFpEF	HFpEF	HFpEF	HFpEF
NSTEMI (Yes/No)	Yes	No	No	No
STEMI (Yes/No)	No	No	No	Yes
Hypotension (Admission BP, mmHg)	No (Normal)	Yes (75/52)	Yes (106/75)	Yes (72/37)
Cancer History				
Cancer Type	None	Renal	Lung	None
Adrenal Metastasis	No	Yes	Yes	No
Adrenal tuberculosis	No	No	No	Yes
Cancer Therapy	None	Axitinib	Liposomal paclitaxel, cisplatin, bevacizumab, envafolimab	None
Radiotherapy	No	No	Yes	No
Adrenal Function				
Type of Adrenal Insufficiency	Secondary	Primary	Primary	Primary
Cortisol (µg/dl)	1.1 (Day 5)	2.18 (Day 3)	2.55 (Pre-admission)	4.14 (Day 5)
ACTH (pg/mL)	11.64 (Day 5)	13.51 (Day 3)	142.43 (Pre-admission)	2,868.23 (Day 5)
Aldosterone (pg/mL)	55.75 (Day 5)	19.11 (Day 3)	–	12.93 (Day 5)
Renin (µIU/mL)	8.18 (Day 5)	15.32 (Day 3)	–	54.87 (Day 5)
Laboratory Findings (Admission)				
Troponin I (ng/mL)	0.259 (Day 6)	0.025	<0.012	0.133 (Day 3)
BNP (pg/mL)	–	518.93	619.13	–
NT-proBNP (pg/mL)	8,230 (Day 6)	–	–	1,010(Day 3)
Serum Sodium (mmol/L)	120	128	136	136
Serum Potassium (mmol/L)	4.1	4.4	3.6	4.4
Serum Glucose (mmol/L)	6.0	5.8	7.7	3.5
Creatinine (µmol/L)	42.7	342.1	73.9	175
Echocardiography (Initial)				
EF (%)	50 (Day 8)	55 (Day 2)	54 (Day 2)	60 (Day 3)
LA (mm)	49 (Day 8)	39 (Day 2)	37 (Day 2)	35 (Day 3)
LV (mm)	44 (Day 8)	50 (Day 2)	55 (Day 2)	47(Day 3)
CO (L/min)	3.9 (Day 8)	4.6 (Day 2)	4.5 (Day 2)	4.5(Day 3)
Echocardiography (1-Month Follow-Up)				
EF (%)	53	55	56	62
LA (mm)	44	35	36	34
LV (mm)	42	45	49	47
CO (L/min)	4.0	4.6	4.6	4.6
Outcomes				
Pre-Discharge Troponin I (ng/mL)	0.03	–	–	0.034
Pre-Discharge BNP (pg/mL)	163.91	108.06	161.34	287.63
1-Month BNP (pg/mL)	117.3	20.36	65.8	177.24

HF, heart failure; HFpEF, heart failure with preserved ejection fraction; HFrEF, heart failure with reduced ejection fraction; NSTEMI, non-ST-segment elevation myocardial infarction; STEMI, ST-segment elevation myocardial infarction; BP, blood pressure; ACTH, adrenocorticotropic hormone; BNP, B-type natriuretic peptide; NT-proBNP, N-terminal pro-B-type natriuretic peptide; EF, ejection fraction; LA, left atrium; LV, left ventricle; CO, cardiac output; IVS, interventricular septum; EDV, end-diastolic volume; RA, right atrium; RV, right ventricle; PA, pulmonary artery; PAP, pulmonary artery pressure.

### Patient characteristics and clinical presentation

The cohort consisted of three males and one female, aged 41–77 years (mean: 61 years). All had HFpEF (initial EF 50%–60%). Two patients (Cases 1 and 4) presented with myocardial infarction (MI)—one with non-ST-segment elevation MI (NSTEMI; peak troponin I 0.259 ng/mL) and one with ST-segment elevation MI (STEMI; peak troponin I 0.133 ng/mL)—confirmed by ECG and angiography showing no obstructive coronary disease as the primary HF cause. Two patients (Cases 2 and 3) had malignancies with adrenal metastasis (renal and lung, respectively) and prior cancer therapies (e.g., chemotherapy, radiotherapy). Hypotension was noted in three patients (Cases 2, 3, and 4; admission systolic BP 72–106 mmHg). Laboratory findings at admission revealed hyponatremia (serum sodium 120–136 mmol/L), low cortisol (1.1–4.14 µg/dl), and elevated ACTH in primary adrenal insufficiency cases (142.43–2,868.23 pg/mL). Initial BNP ranged from 518.93–619.13 pg/mL, and NT-proBNP from 1,010–8,230 pg/mL (Table 1).

### Treatment and outcomes

All patients received hormone replacement therapy (initial IV methylprednisolone 20–40 mg, followed by oral prednisone 7.5–22.5 mg/day) alongside HF management (e.g., diuretics, inotropes as needed). Symptoms (dyspnea, nausea, hypotension) resolved within 3–14 days. Pre-discharge, BNP decreased to 108.06–287.63 pg/mL, and troponin I normalized (0.03–0.034 ng/mL where measured). Echocardiography showed a mean EF increase of 1.75% (range: 0%–3%) by one-month follow-up (final EF 53%–62%). Left atrial (LA) and left ventricular (LV) dimensions reduced (e.g., LA from 35 to 49 mm to 34–44 mm). At one-month follow-up, BNP further declined to 20.36–177.24 pg/mL, with no symptom recurrence reported (Table 1).

### Case presentations

Case 1: A 75-year-old female with no prior history of adrenal insufficiency presented with dizziness and vomiting, triggered by pneumonia, which precipitated an adrenal crisis due to a suspected Rathke cleft cyst (secondary adrenal insufficiency). She developed NSTEMI and HFpEF (EF 50%) on day 6. Prednisone and HF therapy improved EF to 53% and BNP from 8,230 pg/mL (NT-proBNP) to 117.3 pg/mL at one month.

Case 2: A 77-year-old male with no prior history of adrenal insufficiency presented with nausea and hypotension (BP 75/52 mmHg), with adrenal crisis likely triggered by adrenal metastasis from renal cancer (primary adrenal insufficiency). No infectious precipitant was identified. HFpEF (EF 55%) responded to methylprednisolone and prednisone, with BNP falling from 518.93 pg/mL to 20.36 pg/mL at one month.

Case 3: A 51-year-old male with a known history of primary adrenal insufficiency(discontinued prednisone without medical consultation), presented with fever, dyspnea, and concurrent pneumonia, which precipitated an adrenal crisis. HFpEF (EF 54%) improved with steroids and antibiotics; BNP dropped from 619.13 pg/mL to 65.8 pg/mL at one month.

Case 4: A 41-year-old male with no prior history of adrenal insufficiency presented with abdominal pain, diarrhea, hypotension (BP 72/37 mmHg), and ST-segment elevation myocardial infarction (STEMI), with adrenal crisis triggered by active tuberculosis affecting the adrenal glands (primary adrenal insufficiency). HFpEF (EF 60%) and MI resolved with steroids and HF therapy; EF rose to 62%, and BNP reached 177.24 pg/mL at one month.

## Discussion

This case series identifies adrenal crisis as a rare but reversible etiology of heart failure with preserved ejection fraction (HFpEF), offering insights from four patients (three males, one female; aged 41–77 years) diagnosed between January 2022 and January 2025. The cohort presented diverse clinical features: two cases exhibited myocardial infarction (one NSTEMI, one STEMI), two had adrenal metastasis from malignancies (renal and lung), and one had adrenal tuberculosis, reflecting a spectrum of underlying diagnoses. All patients demonstrated marked improvement following hormone replacement therapy and HF management, with symptom resolution within 3–14 days, a mean ejection fraction (EF) increase of 1.75% (range: 0%–3%), and B-type natriuretic peptide (BNP) levels decreasing from an initial range of 518.93–619.13 pg/mL to 108.06–287.63 pg/mL pre-discharge, and further to 20.36–177.24 pg/mL at one-month follow-up. These findings extend the clinical spectrum of adrenal crisis beyond its typical presentation of hypotension and shock, suggesting a potential role as a reversible HFpEF precipitant, including in oncologic patients with adrenal involvement or prior cancer therapies. Epidemiological studies suggest that adrenal insufficiency is relatively common in patients with adrenal metastases, with up to 50% of those with bilateral involvement developing adrenal insufficiency, though adrenal crisis-induced HFpEF remains rare (7, 8). Our findings, while preliminary due to the small sample size, highlight the importance of considering this etiology in diverse patient populations, including those with malignancy.

The mechanisms linking adrenal crisis to HFpEF in this series involve multiple physiological disruptions driven by acute adrenal insufficiency. Cortisol deficiency, ranging from 1.1–4.14 µg/dl across all cases, impairs vascular tone by reducing catecholamine sensitivity and compromises myocardial contractility, contributing to diastolic dysfunction—a hallmark of HFpEF—rather than the systolic failure typical of HFrEF (10). This is compounded by aldosterone deficiency (12.93–55.75 pg/mL in Cases 1, 2, and 4), which disrupts sodium and water balance, leading to volume depletion and hyponatremia (120–136 mmol/L in all cases), thereby reducing preload and exacerbating elevated filling pressures characteristic of HFpEF (12). Our data support this mechanism, as all four patients with HFpEF (initial EF 50%–60%) showed rapid symptom resolution within 3–14 days, a mean EF increase of 1.75% (range: 0%–3%), and BNP reduction from 518.93–619.13 pg/mL to 20.36–177.24 pg/mL at one-month follow-up following steroid therapy, suggesting that acute systemic stressors (e.g., cortisol deficiency, inflammation) drive HFpEF in this context rather than chronic remodeling seen in HFrEF. During screening, one patient with HFrEF was identified but excluded, as their presentation was not consistent with the study's focus on HFpEF, reinforcing that adrenal crisis may preferentially precipitate HFpEF in this cohort, potentially including cases with cancer-related adrenal dysfunction (13). In Cases 1 and 4, myocardial infarction (troponin I 0.133–0.259 ng/mL) likely reflects hypoperfusion-induced ischemic stress, further amplifying cardiac dysfunction in patients with malignancy or other underlying conditions.

Our findings extend the understanding of adrenal crisis by emphasizing its role as a trigger for HFpEF, contrasting with prior reports that predominantly associate it with HFrEF or hemodynamic collapse. Unlike studies such as Wang et al. (2), which described cardiomyopathy with tertiary adrenal insufficiency manifesting as refractory HFrEF and shock, this series uniquely documents HFpEF (EF 50%–60%) and myocardial infarction (NSTEMI in Case 1, STEMI in Case 4) as primary presentations, with rapid recovery following steroid therapy. The association with cancer therapies—such as cisplatin, bevacizumab, and envafolimab in Case 3, or axitinib in Case 2—further distinguishes our cases from single-case reports of adrenal crisis mimicking cardiogenic shock (14). This synergy between therapy-related adrenal insufficiency and HFpEF suggests a novel clinical pattern beyond isolated cardiotoxicity from anthracyclines or checkpoint inhibitors (15, 16), underscoring a compounded risk in oncologic patients that warrants broader recognition compared to the hemodynamic focus of earlier literature.

The management of HFpEF in our cohort relied on hormone replacement therapy and standard HF treatments, with no requirement for mechanical circulatory support (MCS) devices such as intra-aortic balloon pump (IABP), Impella, or extracorporeal membrane oxygenation (ECMO). The rapid resolution of symptoms (within 3–14 days) and preserved ejection fraction (EF 50%–60%) in all cases, coupled with the absence of severe cardiogenic shock as defined by the Society for Cardiovascular Angiography and Interventions (SCAI) classification, likely obviated the need for such interventions (17). In more severe presentations of adrenal crisis with cardiogenic shock, devices like IABP, which reduces afterload and enhances coronary perfusion, or Impella, which provides direct ventricular unloading and greater hemodynamic support, could be considered. The choice between these devices would depend on the degree of hemodynamic compromise, with Impella potentially preferred in SCAI Stage C-E shock due to its superior left ventricular support (17). Future studies exploring adrenal crisis with more profound cardiovascular collapse could evaluate the role of MCS devices to optimize management in such scenarios.

The role of additional diagnostic markers, such as lactate levels, and invasive procedures, like right heart catheterization (RHC), in evaluating HFpEF secondary to adrenal crisis warrants consideration. Elevated lactate levels, indicative of tissue hypoperfusion, could provide insights into the severity of cardiovascular compromise and treatment response in adrenal crisis, particularly in cases with hypotension or myocardial infarction, as observed in three of our patients (Cases 2, 3, and 4). Similarly, RHC could elucidate right heart function and pulmonary pressures, potentially clarifying the contribution of preload reduction to HFpEF in this context. In our series, lactate levels were not routinely measured, and RHC was not performed, as clinical management focused on rapid steroid therapy and non-invasive monitoring (e.g., echocardiography, BNP). Additionally, none of our patients required mechanical circulatory support, such as left ventricular assist devices, due to the preserved ejection fraction (EF 50%–60%) and rapid symptom resolution with hormone replacement. Future studies incorporating serial lactate measurements and RHC could enhance understanding of hemodynamic and metabolic dynamics in this rare HFpEF etiology, particularly in oncologic patients with compounded risk factors.

While adrenal crisis appears central to HFpEF in this series, alternative explanations and study limitations must be considered to contextualize our findings. Cardiotoxic effects of cancer therapies, such as cisplatin and bevacizumab in Case 3, could independently contribute to myocardial dysfunction, given their known associations with endothelial damage and heart failure (11, 15); however, the rapid clinical response to steroids across all cases suggest adrenal insufficiency as the primary driver, with therapy-related effects as potential amplifiers. Limitations include the small sample size (*n* = 4), which restricts generalizability, and the retrospective design, which precludes causal confirmation. Additionally, lactate levels were not routinely measured, precluding analysis of tissue hypoperfusion trends during treatment, and right heart catheterization was not performed, limiting insights into right heart function or pulmonary pressures. None of the patients required left ventricular assist support, as their HFpEF responded to medical management, but such data could be relevant in more severe cases. Furthermore, mechanical circulatory support devices, such as IABP or Impella, were not utilized, as the preserved ejection fraction and rapid response to medical therapy did not warrant their use; however, such interventions could be relevant in cases with more severe cardiogenic shock. The one-month follow-up limits insights into long-term outcomes, such as HFpEF recurrence or adrenal function recovery, while the heterogeneity of etiologies (e.g., tuberculosis in Case 4 vs. metastasis in Cases 2 and 3) complicates a unified phenotype. Future prospective studies with larger cohorts, extended follow-up, and biomarker profiling (e.g., cytokines, cortisol dynamics) are needed to disentangle confounders and establish prevalence and mechanisms.

The reversibility of HFpEF induced by adrenal crisis, as demonstrated by symptom resolution and a mean EF improvement of 1.75% following prompt steroid therapy (methylprednisolone or prednisone) in all four cases, underscores the critical need for early recognition in clinical practice. This is particularly relevant for oncologic patients with adrenal metastasis or prior cancer therapies, who may present with atypical HFpEF or myocardial infarction, highlighting a vulnerable population at the intersection of cardiology and endocrinology. Rather than advocating routine adrenal screening for all at-risk patients, which requires validation from larger studies, we suggest clinicians consider adrenal function testing (e.g., morning cortisol, ACTH) in cancer patients with unexplained HFpEF, especially post-therapy, to facilitate timely intervention. Multidisciplinary collaboration among cardiologists, endocrinologists, and oncologists is essential to optimize diagnosis and management. While preliminary, these findings advocate for heightened awareness and further investigation to refine our understanding of HFpEF etiologies and improve outcomes in this complex cohort.

## Data Availability

The original contributions presented in the study are included in the article/Supplementary Material, further inquiries can be directed to the corresponding author.
